# Histone acylation marks respond to metabolic perturbations and enable cellular adaptation

**DOI:** 10.1038/s12276-020-00539-x

**Published:** 2020-12-11

**Authors:** Chanhee Jo, Seokjae Park, Sungjoon Oh, Jinmi Choi, Eun-Kyoung Kim, Hong-Duk Youn, Eun-Jung Cho

**Affiliations:** 1 School of Pharmacy, Sungkyunkwan University, Suwon, Gyeonggi-do 440-746 Republic of Korea; 2 Department of Brain and Cognitive Sciences, Daegu Gyeongbuk Institute of Science and Technology, Daegu, 42988 Republic of Korea; 3 Neurometabolomics Research Center, Daegu Gyeongbuk Institute of Science and Technology, Daegu, 42988 Republic of Korea; 4 National Creative Research Center for Epigenome Reprogramming Network, Seoul National University College of Medicine, Seoul, 03080 Republic of Korea

**Keywords:** Acetylation, Chromatin remodelling

## Abstract

Acetylation is the most studied histone acyl modification and has been recognized as a fundamental player in metabolic gene regulation, whereas other short-chain acyl modifications have only been recently identified, and little is known about their dynamics or molecular functions at the intersection of metabolism and epigenetic gene regulation. In this study, we aimed to understand the link between nonacetyl histone acyl modification, metabolic transcriptional regulation, and cellular adaptation. Using antibodies specific for butyrylated, propionylated, and crotonylated H3K23, we analyzed dynamic changes of H3K23 acylation upon various metabolic challenges. Here, we show that H3K23 modifications were highly responsive and reversibly regulated by nutrient availability. These modifications were commonly downregulated by the depletion of glucose and recovered based on glucose or fatty acid availability. Depletion of metabolic enzymes, namely, ATP citrate lyase, carnitine acetyltransferase, and acetyl-CoA synthetase, which are involved in Ac-CoA synthesis, resulted in global loss of H3K23 butyrylation, crotonylation, propionylation, and acetylation, with a profound impact on gene expression and cellular metabolic states. Our data indicate that Ac-CoA/CoA and central metabolic inputs are important for the maintenance of histone acylation. Additionally, genome-wide analysis revealed that acyl modifications are associated with gene activation. Our study shows that histone acylation acts as an immediate and reversible metabolic sensor enabling cellular adaptation to metabolic stress by reprogramming gene expression.

## Cellular adaptation: chromosomal protein modification acts as metabolic stress sensor

Tracking the modification of a protein essential to chromosome structure could indicate the metabolic state of cells. Histone proteins provide structural support for chromosomes, and their modification influences metabolic signaling and gene expression. One possible modification adds an acyl group to the histone (acylation). Eun-Jung Cho at Sungkyunkwan University, Suwon, South Korea, and co-workers explored acylation of histone H3K23 under specific metabolic challenges, including reduced availability of glucose and metabolic enzymes. Mammalian cells rapidly alter gene expression in response to nutrient availability, enabling them to adapt under stress. The team found that H3K23 modifications were directly linked with nutrient availability and metabolic enzyme levels. H3K23 acylation specifically reprogrammed gene expression under stress conditions, suggesting that histone acylation is part of a critical sensor system that helps cells adapt to stress.

## Introduction

The posttranslational modification of histone proteins plays a crucial role in the regulation of a wide range of biological processes^1^. Histone lysine (K) acetylation is the most prevalent modification of chromatin, which is reversibly modulated by histone acetyltransferases (HATs) and histone deacetylases (HDACs). Histone acetylation has been intimately linked to cellular metabolism because of its sensitivity to the abundance of acetyl-coenzyme A (Ac-CoA)^2^. Ac-CoA is a central metabolic intermediate produced primarily from glycolysis and various metabolic pathways. It additionally serves as a substrate for histone acetylation at the interface of metabolism and epigenetic regulation of gene transcription^3^. Fluctuating levels of Ac-CoA have a profound effect on the catalytic activity of HATs because many HATs have a relatively high Kd toward Ac-CoA^4^. Therefore, glucose availability, which provides abundant Ac-CoA, is important for histone acetylation.

Metabolic enzymes, especially those that supply Ac-CoA, also play critical roles in the maintenance of histone acetylation. The intracellular Ac-CoA pool is separated into a mitochondrial and a nucleocytosolic fraction by lipid membranes. Recent studies have demonstrated that major metabolic enzymes, such as ATP citrate lyase (ACLY) and carnitine acetyltransferase (CRAT), which convert citrate or mitochondrial acetate to cytosolic Ac-CoA, and acetyl-CoA synthetase 2 (ACSS2), which produces Ac-CoA from acetate, all contribute to the nucleocytosolic Ac-CoA pools and affect histone acetylation states^5–7^. These studies suggest that both the nutrient environment and the activity of metabolic enzymes influence histone acetylation dynamics and subsequent transcriptional activity through modulation of Ac-CoA pools.

More recently, short-chain acyl modifications of histone lysine residues other than acetylation have been identified by mass spectrometry (MS)-associated proteomic analysis^8,9^. These modifications include propionylation, butyrylation, hydroxybutyrylation, crotonylation, malonylation, succinylation, and glutarylation^8,9^. These modifications have a structural similarity with acetylation and are regulated by known HATs and HDACs^10–12^, raising the excellent question: Are they also implicated in metabolic cross talk and epigenetic regulation of gene expression? Indeed, histone propionylation, butyrylation, and crotonylation are distributed in the genome in a pattern that closely resembles that of their acetylation counterparts, suggesting similar and/or distinct roles in gene transcription^13–15^. Histone butyrylation has been suggested to stimulate transcription in competition with acetylation during spermatogenesis^16^. Site-specific histone propionylation at H3K23 and global histone crotonylation were reported to increase during leukemia cell differentiation and mouse spermatogenesis^8,17^. In agreement with this finding, p300-dependent crotonylation of H3K18 was associated with gene activation^15^. Hydroxybutyrylated histone was identified as a mark of active genes associated with starvation-responsive metabolic pathways^18^. However, despite recent advances in the investigation into epigenetic functions of histone acylation, it remains unclear whether histone acylation relays any metabolic information that affects gene regulation or plays any role in the epigenetic adaptation to metabolic changes to maintain cellular homeostasis.

In this study, we aimed to understand the relationship between cellular metabolic status and histone acylation states. Histone acylation was dynamically regulated upon various metabolic perturbations, including glucose deprivation or knockdown of metabolic enzymes that directly affect pools of Ac-CoA and short-chain acyl-coenzyme A (SCA-CoA). By analyzing the specific and dynamic acylation levels of H3K23 as an epigenetic marker, we found that the epigenome actively responds to metabolic changes to alleviate metabolic stress by genome-wide remodeling of histone acylation.

## Materials and methods

### Culture conditions and fetal bovine serum (FBS) dialysis

C2C12 cells were obtained from American Type Culture Collection and cultivated as previously described^19^. Fetal bovine serum (FBS) (Gibco, Cat# 2640-0440) was dialyzed against 150 mM NaCl using 10,000 Dalton molecular weight cutoff dialysis tubing (CelluSep #1-1050-40) to remove residual metabolites^20,21^. C2C12 myotube cells were cultivated in high glucose (25 mM) DMEM (Gibco, cat #11965-092) supplemented with 2% (v/v) dialyzed horse serum. To induce glucose starvation, the medium was replaced with glucose-deficient (0 mM) DMEM (Gibco, cat #11966-025) supplemented with dialyzed serum. For the fatty acid supplementation experiment, 200 μM oleic acid conjugated with BSA (Sigma, cat #O3008) or BSA alone (Millipore, cat #82-002-2) was added to the medium. L-Carnitine (1 mM) was added to media for 24 h prior to treatment with oleic acid/BSA to facilitate utilization of fatty acids^22^.

### Immunoprecipitation (IP)

Cells were harvested, lysed, sonicated, and centrifuged to remove debris to prepare chromatin solution. Overnight incubation was carried out with 2 μg of each specific antibody and A/G agarose beads (GE, 17-0618-01 and 17-5280-01) at 4 °C. On the following day, beads were washed three times, and precipitated proteins were analyzed with immunoblot analysis. The antibodies used in this study are listed in Supplementary Table 1.

### Immunoblot analysis

Cells were lysed and sonicated. Equal amounts of protein were separated by SDS-PAGE and transferred onto NC membranes. The membranes were blocked with 1% BSA in TBST [20 mM Tris, 150 mM NaCl, 0.1% (w/v) Tween 20] for 1 h and incubated overnight with primary antibodies. After washing several times with TBST, secondary antibodies were added and incubated for 1 h at room temperature. Signals were visualized by the ECL detection kit (AbFrontier, cat #LF-QC0103).

### Generation of immunopurified polyclonal H3K23 acyl-specific antibodies

Immunopurified rabbit polyclonal anti-H3K23Bu, anti-H3K23Cr, and anti-H3K23Pr antibodies were generated by PTM Biolab. Briefly, rabbits (3 animals for each antibody type) were immunized with H3K23-marked peptides of synthetic QLAT(butyryl)**K**AARKC, QLAT(crotonyl)**K**AARKC, and QLAT(propionyl)**K**AARKC, corresponding to residues encompassing K23 of human/mouse histone H3. The antibodies are purified by protein G-conjugated agarose column followed by affinity chromatography, each with a peptide-conjugated column. The antibodies were monitored and validated by ELISAs and dot blot analysis with immunogen.

### RNA interference (RNAi)

siRNAs were transfected using Lipofectamine RNAiMax^®^ (Invitrogen) according to the manufacturer’s instructions. siRNAs were designed using the siDESIGN tool (https://dharmacon.horizondiscovery.com) and purchased from Bioneer (Korea). Cells were harvested 48–96 h after siRNA transfection. The siRNA sequences are shown in Supplementary Table 2.

### RNA extraction and quantitative real-time PCR (qRT-PCR)

Total RNA was purified using NucleoSpin^®^ (Macherey Nagel, 740955.250) according to the manufacturer’s instructions. RNA purity and integrity were evaluated using an ND-1000 spectrophotometer (NanoDrop, Wilmington, USA) and an Agilent 2100 Bioanalyzer (Agilent Technologies, Palo Alto, USA). A total of 2 μg of RNA was reverse-transcribed with a cDNA synthesis kit (Thermo, K1632). Quantitative real-time PCR was performed using a CFX96 System (Bio-Rad, Hercules, CA, United States). PCR amplification was carried out using KAPA SYBR FAST Master Mix (KAPA Biosystem, Wilmington, MA, United States), and relative quantification was calculated by the 2^−ΔΔCT^ method. The primers used for RT-qPCR are listed in Supplementary Table 2.

### Chromatin immunoprecipitation (ChIP)

C2C12 cells were cross-linked with 1% formaldehyde. Cross-linking was quenched by the addition of glycine. Nuclear fractions were sonicated to produce DNA fragments ranging from 200 to 1000 bp using a Bioruptor (Diagenode) sonicator. After centrifugation, antibodies and beads were added to the chromatin solution and incubated overnight at 4 °C. After de-cross-linking, the DNA was purified using a PCR purification kit (Qiagen, Hilden, Germany).

### ChIP sequencing (ChIP-seq) and data analysis

ChIP samples were QC-tested for sample integrity and purity using an Agilent Bioanalyzer 2100 (Agilent Technologies, Inc.). ChIP libraries were prepared using QC-passed ChIP-ed DNA samples according to the DNBseq ChIP-Seq library preparation protocol. The PCR products were purified and selected with the Agencourt AMPure XP-Medium kit. The concentration and quality of DNA with adapters were determined using the Qubit ssDNA kit. The library was amplified using phi29 to make DNA nanoballs (DNBs). The DNBs were loaded into a patterned nanoarray, and single-end 50 bp reads were generated by combinatorial probe-anchor synthesis (cPAS) using DNBSEQ-T7. For the data analysis, the reads were mapped with Bowtie 2.2.5 using mm10 as the reference genome. Uniquely mapped reads were filtered using SAMtools 1.2. MACS 2.1.1 was used to call significant peaks (*q*-value < 0.05). The coverage was normalized with the total read numbers within the combined H3K23Ac narrow peaks. Size factors were calculated by DESeq2. Significantly differentially regulated peaks were identified using EdgeR (dispersion = 0.05 and *p*-value <0.05).

### Microarray, RNA-sequencing, and data analysis

Total RNA was amplified and purified using the TargetAmp-Nano Labeling Kit for Illumina Expression BeadChip (Epicentre, Madison, USA) to yield biotinylated cRNA and each was hybridized to MouseRef-8 v2.0 Expression BeadChip (Illumina, Inc., San Diego, USA). Detection of the array signal was carried out using Amersham fluorolink streptavidin-Cy3 (GE Healthcare Bio-Sciences, Little Chalfont, UK). Raw data were extracted using Illumina GenomeStudio v2011.1 software, Gene Expression Module v1.9.0. For RNA sequencing, library construction was performed using a QuantSeq 3′ mRNA-seq Library Prep Kit (Lexogen, Austria). High-throughput sequencing was performed as single-end 75 bp sequencing using NextSeq 500 (Illumina, USA). QuantSeq 3′ mRNA-seq reads were aligned using Bowtie2. DEGs were determined based on counts from unique and multiple alignments using coverage in Bedtools. The read count data were processed based on the quantile normalization method using the EdgeR package. Gene classification was based on the DAVID (http://david.abcc.ncifcrf.gov) and Medline (http://www.ncbi.nlm.nih.gov) databases. Heat map visualizations were constructed with MEV software (v 4.9.0).

### Ac-CoA, Acyl-CoA, and CoA analyses by LC–MS

Cells were extracted in 500 μl of methanol:water (8:2, v:v) containing an internal standard (100 nM acetyl-1,2-^13^C_2_-CoA). Samples were sonicated and centrifuged. The supernatant was transferred to new tubes and evaporated. The samples were reconstituted in 100 μl of initial mobile phase (20 mM ammonium acetate in water). The reconstituted solution was filtered, and an aliquot (20 μl) was injected into the LC–MS system. Metabolites were analyzed using an Agilent 1290 Ultra-Performance Liquid Chromatography system coupled with an Agilent 6490 triple quadrupole mass spectrometer (Agilent LC-QQQ-MS/MS, Agilent Technologies) in positive electrospray ionization (ESI) mode. The system was operated in multiple reaction monitoring mode using individually optimized collision energy. Chromatographic separation was achieved on an Agilent Poroshell EC-C18 column (100 mm × 2.1 mm, 2.7 μm). The data processing for both qualitative and quantitative analyses was performed using Agilent MassHunter software (Agilent Technologies).

### GC–MS metabolite analysis

Cells were extracted with 800 μl of methanol:chloroform:water, 2:1:1 (v:v) containing an internal standard (20 nM 2-isopropylmalic acid and D2-oleic acid). Samples were sonicated and centrifuged, and the upper aqueous phases were transferred to a glass test tube, and the metabolites such as those from glycolysis, TCA cycle, and pentose phosphate pathway (PPP) (group 1) were analyzed. The lower organic phase was transferred to new tubes for the analysis of fatty acids (group 2). All samples were evaporated and derivatized by using 20 mg/ml methoxyamine hydrochloride in pyridine coupled with MSTFA (group 1, Sigma, 69479) and BCl_3_-methanol (12% w/w, group 2, Sigma, 33033) according to the manufacturer’s instructions. All samples were injected into the GC–MS system. Metabolites were analyzed using an Agilent 7000B gas chromatography system coupled with a 7000 C tandem mass spectrometric detector (Agilent GC-QQQ-MS/MS, Agilent Technologies) and equipped with an ultra HP-5 ms capillary column (30 m × 0.25 μm, i.d., 0.25 μm film thickness, Agilent J&W Scientific). The data processing for both qualitative and quantitative analyses was performed using Agilent MassHunter software (Agilent Technologies).

### Animal study

For starvation experiments, 3-week-old C57BL/6 J mice were purchased from Central Lab Animal Inc. and maintained on a normal diet for 5 weeks. The mice were randomly allocated to the following three experimental groups: control group (*n* = 4), fed ad libitum for 24 h; starvation group (*n* = 4), starved for 24 h; refed group (*n* = 5), starved for 24 h followed by 6 h of feeding. The mice were then sacrificed, and the soleus muscles were dissected. The muscle tissue was homogenized and sonicated with a Bioruptor (Diagenode) for 10 min and centrifuged. All animal experiments were approved by the Institutional Animal Care and Use Committee of Sungkyunkwan University (approval no. SKKUIACUC2019-03-22-1).

### Statistical analysis

Statistical analysis was performed using GraphPad Prism V6.0 software (San Diego, CA, USA). The results are presented as the means ± SEM. *p* < 0.05 was considered statistically significant. Significant differences between groups were determined by Student’s *t* test or ANOVA multiple comparisons test.

## Results

### Histone acylation is closely linked to glucose metabolism

**Fig. 1:**
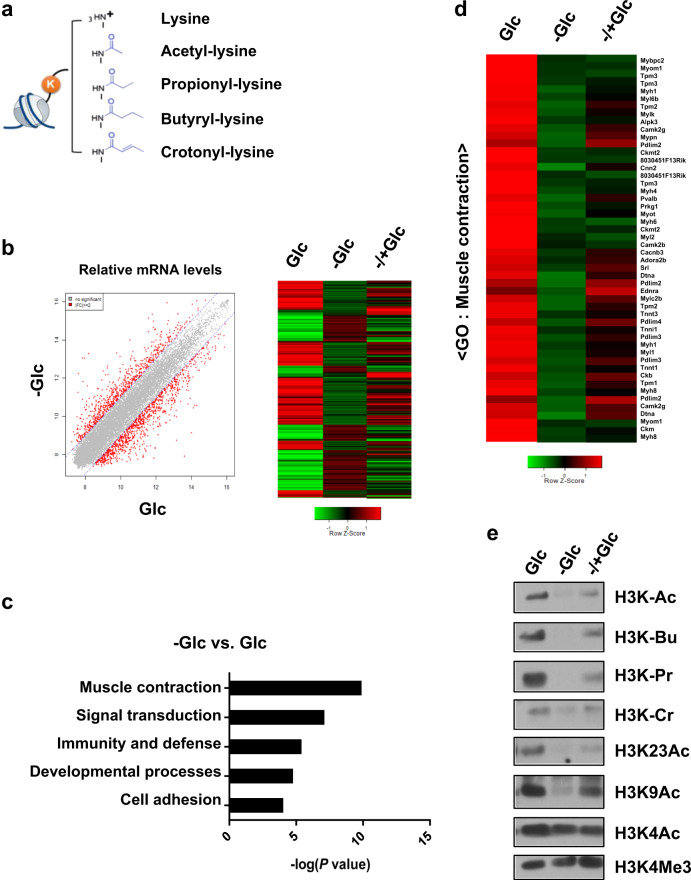
Histone acylation is closely linked to metabolic conditions. **a** Acetylation, propionylation, butyrylation, and crotonylation of lysine residues on histone tails. **b** Microarray data of glucose-deprived C2C12 myotube cells. Left; scatter plot with red dots representing genes with significantly altered expression (FC ≥ 2). Right panel: total heatmap (FC ≥ 2). C2C12 myotube cells were cultured in “Glc”, high-glucose DMEM for 24 h; “-Glc”, no glucose DMEM for 24 h; or “−/+Glc”, no glucose DMEM for 24 h followed by high-glucose DMEM for 12 h. **c** Top 5 DAVID annotated gene ontology groups for significantly altered genes under “-Glc” conditions relative to “Glc” conditions. **d** Heat map of the “muscle contraction” gene set from the microarray dataset. **e** WB with the indicated antibodies was used to identify histone modification.

Various short-chain acyl modifications, such as propionylation (Pr), butyrylation (Bu), and crotonylation (Cr), are considered functional epigenetics marks (Fig. 1a). However, it is not known whether these histone acyl modifications have any metabolic implications. To investigate the link between metabolic changes and epigenetic regulation, we performed microarray experiments with C2C12 myotubes (murine skeletal muscle cells) grown in a standard glucose concentration (25 mM Glc), glucose starved (-Glc, 24 h), or 12 h of glucose following 24 h of glucose starvation (-/+Glc). Our transcriptome analysis showed that a total of 1643 genes were significantly affected by glucose deprivation (934 downregulated, 709 upregulated, |fold change (FC) | ≥ 2), and their expression was partially rescued by glucose addition (Fig. 1b). Gene ontology (GO) analysis showed that genes that rely on high energy consumption in muscle tissue, such as those involved in muscle contraction, were affected the most by glucose deprivation (Fig. 1c, d). Under these experimental conditions, the acylation states of histones were analyzed. The immunoblot analysis showed that global levels of H3 pan-acyl modifications, including H3K-Bu, H3K-Pr, and H3K-Cr, as well as H3K-Ac, were all dramatically downregulated by glucose withdrawal and partially restored by glucose re-addition (Fig. 1e). The glucose sensitivity of H3 lysine residues (H3K23Ac, H3K9Ac, and H3K4Ac) was variable, indicating that each residue has distinct acetylation turnover rates, whereas H3 methylation (H3K4Me3) remained largely unchanged by 24 h of glucose deprivation. These data indicate that, in addition to acetylation, histone butyrylation, crotonylation, and propionylation were reversibly regulated and closely linked to glucose availability.

### H3K23 acylation is reversibly modulated upon metabolic perturbation

**Fig. 2:**
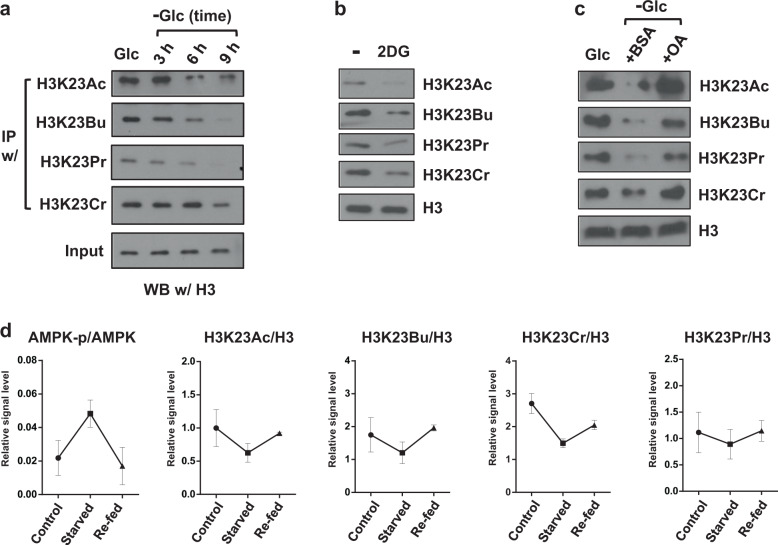
H3K23 acylation is reversibly modulated upon metabolic perturbation. **a** WB of C2C12 myotubes grown under glucose-deprivation conditions. C2C12 myotubes were cultivated in glucose-deficient medium for the indicated period. Anti-H3K23 acylation-specific antibodies were used for protein IP, which were subsequently immunoblotted with anti-H3 antibody. **b** WB of H3K23 modifications using the indicated antibodies with cellular extracts obtained from C2C12 myotubes treated with/without 2DG (25 mM) for 24 h. **c** WB of H3K23 acylation in C2C12 myotubes grown in “Glc”, high glucose-DMEM for 24 h; “BSA”, no glucose DMEM for 24 h with fatty acid-free BSA; or “+OA”, no glucose DMEM for 24 h with oleic acid-conjugated BSA (200 μM oleic acid). **d** Relative quantitation of WB bands representative of the in vivo starvation experiment. “Control” (*n* = 4), fed ad libitum 24 h; “Starved” (*n* = 4), no food for 24 h; “Refed” (*n* = 5), starved for 24 h followed by refeeding for 6 h. Soleus muscles from each mouse were analyzed by WB using acylation-specific antibodies. H3K23 acylation and p-AMPK were normalized to their control counterparts, H3 and AMPK, respectively. The error bars indicate the SEM for each group.

To further understand the link between metabolism and the histone acylome, we focused on the H3K23 residue for the following reasons. H3K23 is a fast turnover site^23^ and is sensitive to an abundance of metabolic substrates^24^. Furthermore, it is variously modified by acetylation, butyrylation, propionylation, and crotonylation^8,25,26^. H3K23 modification might also have a role at the level of cellular phenotype^8,17^. We developed specific antibodies that recognized H3K23 butyrylation (H3K23Bu), propionylation (H3K23Pr), and crotonylation (H3K23Cr) with high specificity and negligible cross-reactivity (Supplementary Fig. S1). We used these antibodies for the IP assay to enrich modified histones from C2C12 cells grown in glucose- or glucose-deficient medium. Immunoprecipitates were analyzed by WB with an anti-H3 antibody. Our WB data showed that H3K23Ac and all three acylated marks were diminished upon glucose withdrawal (Fig. 2a and Supplementary Fig. S2a), consistent with the aforementioned data. The global reduction in H3K23 modification was recapitulated, as indicated by direct immunoblotting of cellular proteins following treatment with 2-deoxyglucose (2DG), a glycolysis inhibitor (Fig. 2b).

Next, we examined the effect of fatty acids on histone acylation. Oleic acid (C18), an even chain fatty acid, is known as an alternative source of Ac-CoA for histone acetylation^27^. In principle, oleic acid can provide SCA-CoA as a metabolic precursor for histone butyrylation (4 C) and crotonylation (4 C) but not propionylation (3 C). However, the immunoblot analysis showed that the H3K23 acylation levels that were affected by glucose deprivation recovered upon cell treatment with BSA-conjugated oleic acid (OA) but not the BSA-only treatment (Fig. 2c). This phenomenon was also confirmed with HepG2 cells, for which the OA treatment was sufficient to restore H3K23 acylation, including propionylation (Supplementary Fig. S2b,c). These data indicate that 1) both glucose and fatty acids can provide a metabolic input for histone acylation; 2) these modifications might result from more complicated metabolic reprogramming than previously thought; and 3) H3K23 is a reliable epigenetic mark that can sense the cellular metabolic situation.

We also analyzed soleus skeletal muscle tissues obtained from mice given ad libitum access to food, mice that were starved (complete fasting for 24 h), and refed mice (6 h of refeeding after 24 h of fasting) using WB analysis with H3K23 antibodies. Phosphorylated AMPK, the active form of AMPK, was used as a starvation marker^28^, while H3 was used for normalization. Consistent with that of the in vitro experiment, our analysis showed that starvation induced deacylation of H3K23, while refeeding led to recovered modification, which were similar to the levels of the mice fed ad libitum (Fig. 2d). Taken together, these data show that histone butyrylation, propionylation, and crotonylation, as well as acetylation, are closely associated with central glucose metabolism.

### Direct perturbation of Ac-CoA pools impacts histone acylation

**Fig. 3:**
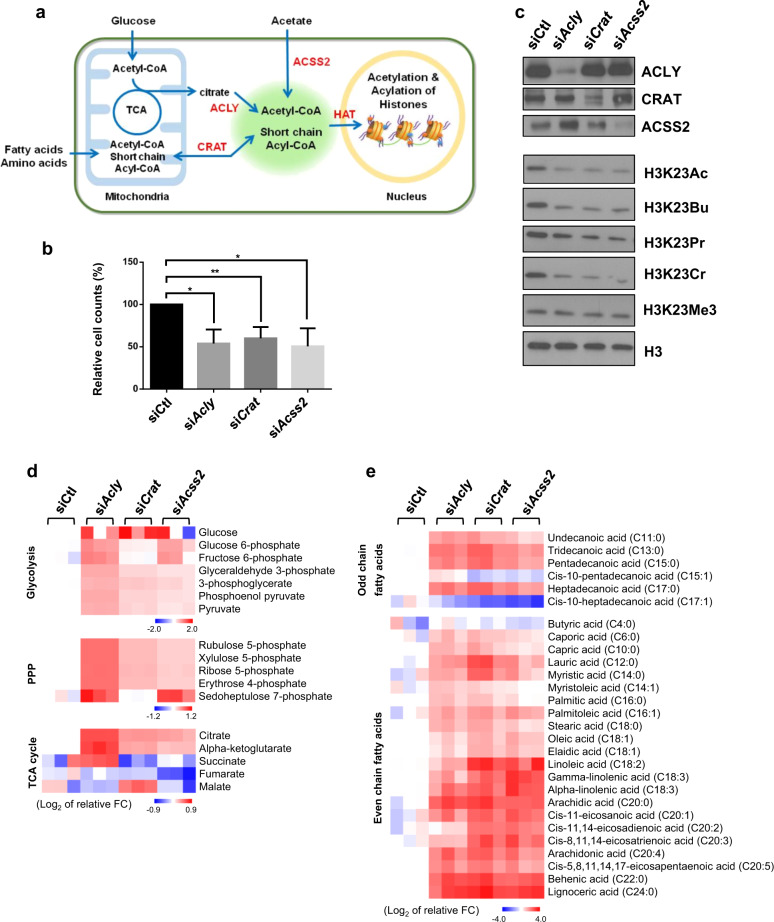
Impact of direct perturbation of Ac-CoA pools on histone acylation. **a** Schematic presentation depicting the flux of metabolites mediated by ACLY, CRAT, and ACSS2, which contributes to Ac-CoA/SCA-CoA pools that support nuclear histone modification. **b** Relative cell count (%) of the indicated siRNA-transfected C2C12 myoblasts compared to the percentage of siCtl. The cells were analyzed 3 days after siRNA treatment. The error bars indicate the SEM (*n* = 5). Statistical significance was analyzed based on comparison to siCtl, *p < 0.05; **p < 0.01. **c** WB of total lysates obtained from C2C12 myoblasts transfected with the indicated siRNAs. **d**–**e** Heat maps of metabolite fold changes. C2C12 myoblasts were transfected with the indicated siRNAs for 72 h, and metabolite abundance was measured using GC–MS; each column indicates an individual sample (*n* = 3). Glycolysis, PPP, and TCA cycle intermediates (**d**) and fatty acids (**e**) are shown.

To directly address the link between metabolic input and the acyl modification of histones, we perturbed the intracellular pools of Ac-CoA/SCA-CoA by knocking down the genes encoding the ACLY, ACSS2, and CRAT enzymes, which are critical for Ac-CoA/SCA-CoA levels. ACLY provides nucleocytosolic Ac-CoA by cleaving citrate derived from mitochondria and plays a critical role in histone acetylation. An alternative source of Ac-CoA is provided by ACSS2 and CRAT; ACSS2 directly catalyzes Ac-CoA from acetate, while CRAT is an important modulator of the Ac-CoA pools in muscle cells (Fig. 3a). ACSS2 and CRAT can also provide SCA-CoA in addition to Ac-CoA. We confirmed that the RNAi-mediated knockdown of *Acly*, *Acss2*, or *Crat* selectively suppressed the expression of their respective targets (Fig. 3c). RNAi-induced knockdown of these enzymes ultimately resulted in a reduction in cell growth (Fig. 3b). Furthermore, it caused a significant global reduction in H3K23Ac, H3K23Bu, H3K23Pr, and H3K23Cr, with no effects on H3K23Me3, in both C2C12 myoblasts (Fig. 3c) and myotubes (Supplementary Fig. S3). Interestingly, even though Ac-CoA is the only product of the enzymatic reaction of ACLY, *Acly* knockdown resulted in a global reduction in H3K23 acylation, indicating that the simultaneous decline in all acyl modification levels might be a common epigenetic response to the perturbation of Ac-CoA pools.

To further understand the metabolic features induced by depletion of metabolic enzymes, we performed comparative metabolome analysis using GC–MS. RNAi-mediated knockdown of *Acly, Acss2*, and *Crat* led to largely similar metabolic profiles compared to the profile of the siRNA control. In particular, knocking down these genes resulted in a significant accumulation of intermediates of glycolysis, PPP, and the TCA cycle, indicating that diminished replenishment of Ac-CoA pools results in incomplete process from glycolysis to ATP production and/or nucleotide synthesis (Fig. 3d). This interruption explains in part why these cells displayed significantly reduced cell proliferation (Fig. 3b). Furthermore, we observed significant accumulation of fatty acids of various chain lengths (Fig. 3e). Similar to previous reports showing that ACLY- or CRAT-deficient cancer cells displayed defective lipid synthesis and an increase in fatty acids^29–31^, our data showed that knocking down *Acly, Acss2*, or *Crat* perturbed lipid metabolism and led to fatty acid accumulation, indicating that metabolic imbalance likely resulted from a shortage of intracellular Ac-CoA pools. Taken together, our results indicate that Ac-CoA levels and histone acylation are closely linked and might elicit cellular metabolic adaptation.

### The cellular levels of Ac-CoA and the Ac-CoA/CoA ratio modulate histone acylation

**Fig. 4:**
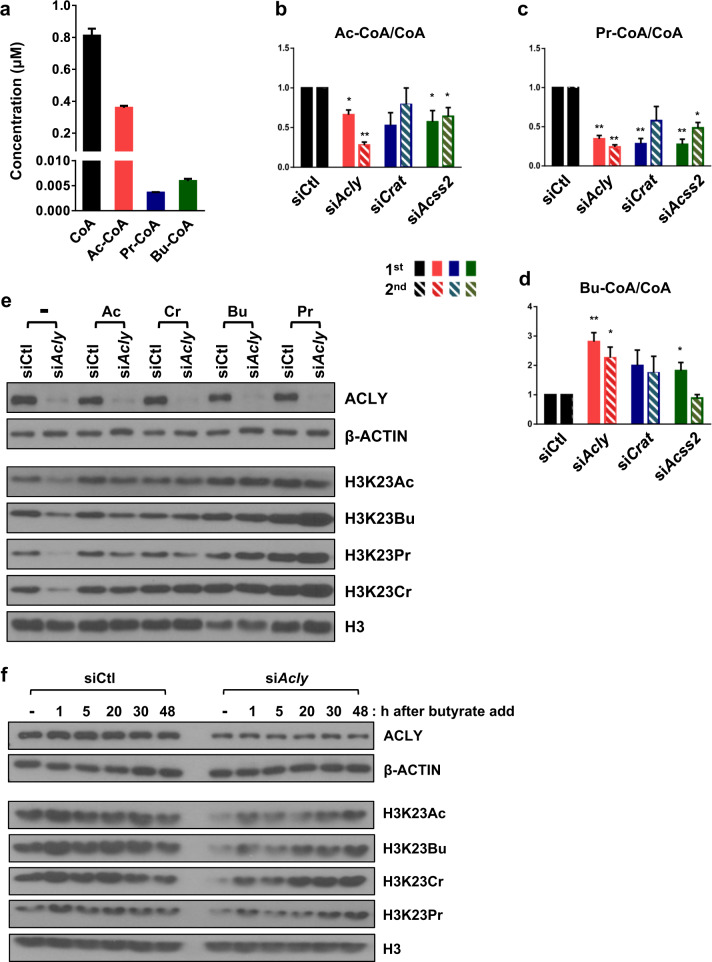
Cellular levels of Ac-CoA and the Ac-CoA/CoA ratio modulate histone acylation. **a** Bar plot of intracellular concentrations of CoA, Ac-CoA, Pr-CoA, and Bu-CoA in C2C12 myoblasts. The relative ratios of Ac-CoA/CoA (**b**), Pro-CoA/CoA (**c**), and Bu-CoA/CoA (**d**) were measured using LC–MS. Metabolic enzymes in C2C12 myoblasts was knocked down with specific siRNAs. Metabolome analysis is indicated in two independent experiments (1^st^, 2^nd^); the error bars indicate the mean SEM (*n* = 3 per group); *p < 0.05; **p < 0.01 compared to siCtl. **e** WB of siRNA-treated C2C12 myoblasts. Each supplement (Ac: acetate, Cr: crotonate, Bu: butyrate, and Pr: propionate) was added to the culture medium (10 mM for 24 h). **f** WB of the siRNA-treated C2C12 myoblasts. Butyrate (100 μM) was added to the medium for the indicated times; *p < 0.05, **p < 0.01.

The enzymatic activity of HATs is largely affected by the relative abundance of Ac-CoA and free CoA (Ac-CoA/CoA ratio) due to product inhibition mediated by CoA^4,32,33^. As *Acly* knockdown had a profound effect on histone acylation, we hypothesized that changes in the Ac-CoA/CoA ratio might be important for HAT activity toward histone butyrylation, crotonylation, and propionylation as well as histone acetylation^10,15,34^. Therefore, we sought to determine empirical amounts of CoA, Ac-CoA, and SCA-CoA and to determine whether their relative ratios were affected by RNAi-mediated knockdown. Our data showed that free CoA and Ac-CoA were the most abundant CoA species in C2C12 cells, while the propionyl-CoA (Pr-CoA) and butyryl-CoA (Bu-CoA) were comparatively very low, and crotonyl-CoA (Cr-CoA) was negligibly detected in this experimental system (Fig. 4a). Further analysis of the knockdown samples showed that depletion of ACLY, CRAT, or ACSS2 commonly resulted in a significant decrease in the Ac-CoA/CoA and Pr-CoA/CoA ratios, while on the contrary, the Bu-CoA/CoA ratio was increased (Fig. 4b–d). These data indicated that depletion of ACLY, ACSS2, or CRAT resulted in a significant decrease in Ac-CoA, impacting the Ac-CoA/CoA ratio, which may subsequently influence histone butyrylation, crotonylation, and propionylation via the alteration of HAT activity. Although the Bu-CoA/CoA ratio was increased as a cellular response to compensate for metabolic stress, it seemed insufficient to independently promote H3K23Bu modification.

To test this hypothesis, we assessed the effect of exogenously supplied acetate, crotonate, butyrate, and propionate on H3K23 acylation. For a comprehensive analysis, we focused on ACLY, the role of which has been extensively studied in histone acetylation in diverse cell types. As anticipated, supplementation with acetate (10 mM) was sufficient to suppress the *Acly* knockdown-mediated reduction in H3K23Ac, H3K23Bu, H3K23Pr, and H3K23Cr, indicating that exogenous acetate can be used to replenish Ac-CoA pools as substrates and for modulating HAT activity (Fig. 4e). Furthermore, supplementation with crotonate, butyrate, or propionate efficiently recovered the diminished H3K23 acyl states (Fig. 4e). However, as butyrate and propionate, but not acetate and crotonate, can act as potent HDAC inhibitors^35^, we cannot rule out the possibility that they exerted their effects via HDAC inhibition. To clarify the mechanism, we used suboptimal concentrations of butyrate (100 μM) as a metabolic supplement. Butyrate at a concentration of less than 0.5 mM (IC_50_ = 1.13 mM) lacks HDAC inhibitory activity and has a known role as a metabolite^35,36^. As shown in Fig. 4f, butyrate supplementation began to restore H3K23 acylation within 1 h. Taken together, our data indicate that metabolic enzymes are critical for the maintenance of the Ac-CoA pools and that the Ac-CoA/CoA ratio is the most critical determinant for global histone acylation and potentially for subsequent gene expression.

### Transcriptome analysis reveals a cohort of genes sensitive to metabolic perturbation

**Fig. 5:**
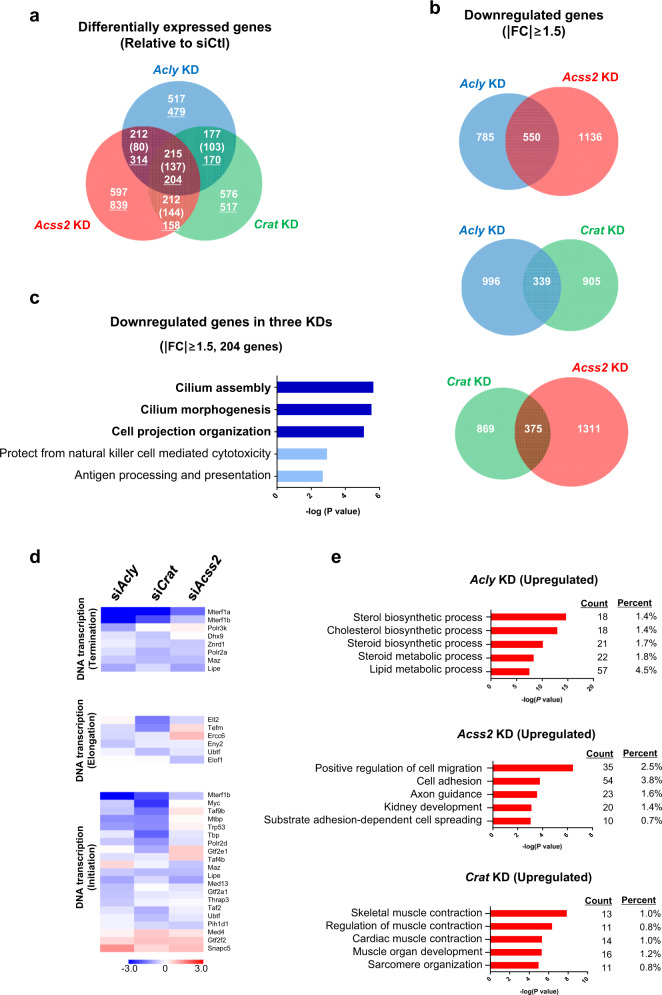
Transcriptome analysis reveals a cohort of genes sensitive to metabolic perturbation. **a** Venn diagram showing differentially expressed genes upon *Acly-*, *Acss2-*, or *Crat*-knockdown in C2C12 myoblasts (relative to the level of siCtl, |FC | ≥ 1.5). Numbers represent the number of commonly upregulated or downregulated (underlined) genes following siRNA treatment. The values in brackets represent gene numbers differently regulated within paired groups. **b** Venn diagram showing the number of downregulated genes from *Acly-*, *Acss2-*, and *Crat*-knockdown C2C12 myoblasts (relative to the level of the siCtl, |FC | ≥ 1.5). **c** GO analysis of 204 commonly downregulated genes (|FC | ≥ 1.5). The top 5 biological functions are shown in order of ascending *p*-values. **d** Heat map of differentially expressed genes associated with DNA transcription (categorized into initiation, elongation, and termination). **e** GO analysis of significantly upregulated genes (|FC | ≥ 1.5). The top five biological functions are shown in order of ascending *p*-value. Each siRNA-mediated knockdown sample was compared with the siCtl.

To gain insight into the transcriptomic response to metabolic perturbation, we performed RNA-seq analysis. A total of 2608, 3112, and 2613 genes were found to be significantly affected by the RNAi-mediated knockdown of *Acly*, *Acss2*, and *Crat*, respectively (|FC | ≥ 1.5) (Fig. 5a and Supplementary Fig. S4a). In contrast to their very similar metabolic profiles, the transcriptome analysis revealed a diverse range of genes from different knockdown backgrounds. Since the most prominent epigenetic response was loss of H3K23 acyl marks, we were particularly interested in downregulated genes that might contribute to the global loss of the H3K23 acylation. Interestingly, a large portion of the downregulated genes significantly overlapped among the knockdown samples (Fig. 5b and Supplementary Fig. S4b). To gain insights into the genomic regions particularly sensitive to Ac-CoA levels, we performed a GO analysis of the functional enrichment with 204 genes that were commonly suppressed by depletion of all three metabolic enzymes. Our analysis highly ranked a cohort of genes associated with cilium assembly, cilium morphogenesis, and cell projection organization (Fig. 5c). These genes are involved in the cell fate of proliferation versus differentiation, depending on energy availability^37–39^. Additionally, we also found that genes associated with RNA synthesis (DNA transcription) were significantly downregulated under all three knockdown conditions (Fig. 5d), which may partially account for the apparent growth defects (Fig. 3b) and accumulated precursors of nucleotide synthesis (Fig. 3d).

Interestingly, in contrast to downregulated genes, many genes involved in diverse but important biological functions were upregulated upon metabolic gene knockdown. For example, genes associated with sterol metabolism (*Acly* knockdown), cell migration and adhesion (*Acss2* knockdown), and muscle contraction (*Crat* knockdown) were upregulated (Fig. 5e), indicating that biological pathways seemingly related to cell–cell fusion and muscle cell differentiation might be turned on. Taken together, these data showed that genes were either suppressed or elevated upon Ac-CoA depletion in a manner that shifted cells away from proliferative growth. This suggests that genome-wide remodeling of histone acylation occurs in response to cellular metabolic demand.

### H3K23 acylation marks are associated with active transcription and undergo remodeling following *Acly* knockdown

**Fig. 6:**
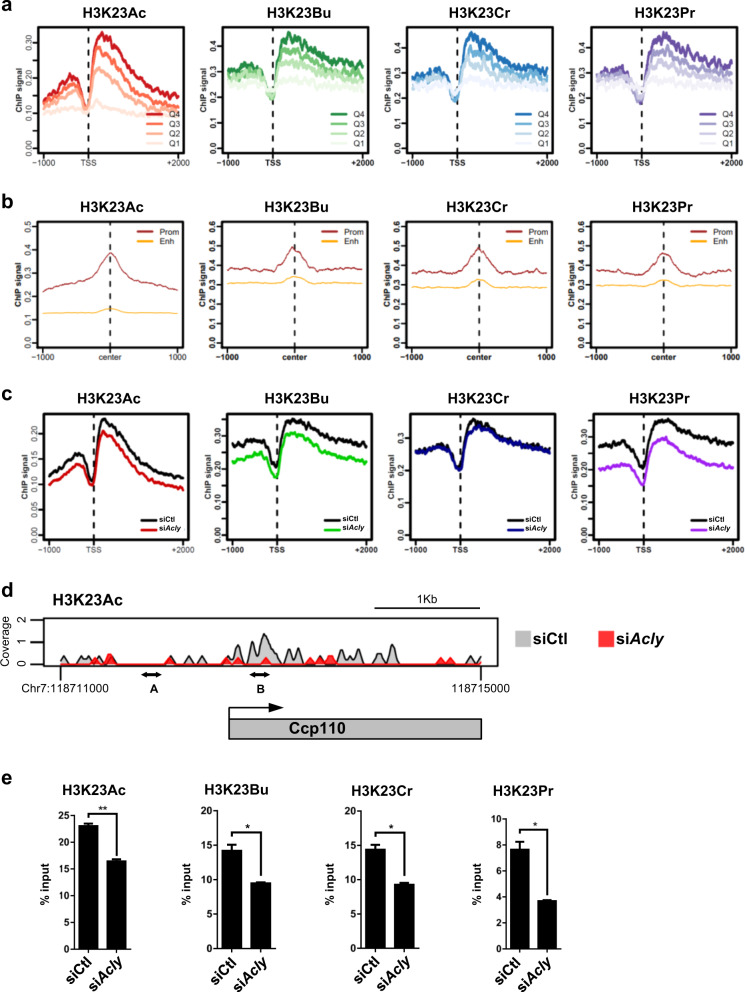
H3K23Ac marks are associated with active transcription and undergo remodeling following *Acly* knockdown. **a** siCtl-transfected C2C12 myoblast cells were analyzed by ChIP-seq with the indicated antibodies. The average ChIP signals of the indicated histone modification were aligned to the TSSs of expressed genes and grouped into four quartiles: Q4, 75–100% (high expression); Q3, 50–75%; Q2, 25–50%; Q1, 0–25% (low expression). The list of differentially expressed genes is from the RNA-seq data. **b** ChIP-seq signals of the indicated H3K23 acyl marks ±1000 bp from the center of the H3K27Ac peaks. The H3K27Ac data was obtained from the GSE37525 dataset. H3K27Ac peaks found within −1000 bp ~ +500 bp of a TSS were classified as promoter peaks (Prom, brown). All the other peaks were classified as putative enhancers (Enh, orange). **c** Metagene profiles of the indicated histone modifications of control or *Acly* knockdown samples were aligned to the TSS of expressed genes. **d** Genome browser view of the H3K23Ac coverage of the *Ccp110* gene locus. **e** Acyl modification of the *Ccp110* promoter region was analyzed by ChIP-qPCR with anti-H3K23 acylation-specific antibodies. The error bars indicate SEM between duplicate experiments; *p < 0.05, **p < 0.01.

To gain insights into the global distribution of H3K23 acylation marks and the genomic regions particularly sensitive to changes in Ac-CoA pools, we performed ChIP-seq and analyzed metagene profiles using genes with read counts greater than 0 from the RNA-seq data (15,748 genes). We observed that H3K23 acylation marks were highly correlated with one another and significantly enriched immediately downstream and upstream of transcription start sites (TSSs), implying that H3K23 acylation is associated with transcriptional regulation (Supplementary Fig. S5). We grouped genes into four quartiles, from Q4 (top 25%) to Q1 (lowest 25%), according to their expression levels. When the average ChIP signals of each H3K23 acyl mark was aligned with similarly grouped genes, we observed that H3K23 acylation had a strong correlation with active transcription (Fig. 6a). H3K27Ac is a representative chromatin mark of both the promoter and enhancer of active genes^40^. Using datasets (GSE37525) published in Blum et al., we identified active regulatory regions marked by H3K27Ac and found that H3K23Ac, H3K23Br, H3K23Pr, and H3K23Cr were more preferentially associated with gene promoters than enhancers, suggesting that these epigenetic marks play roles in promoter regulation (Fig. 6b). Next, to determine the impact of *Acly* knockdown on histone acylation on a genome-wide scale, we compared the ChIP-seq signals of si*Acly* with those of siCtl. *Acly* knockdown resulted in a significant reduction in H3K23Ac, H3K23Bu, and H3K23Pr, but not in H3K23Cr, near the TSS region (Fig. 6c), suggesting that *Acly* knockdown induced H3K23 deacylation at the promoter region. Since our transcriptome analysis revealed genes involved in cilium morphogenesis as primary targets of *Acly* knockdown, we further analyzed the epigenomic states of this category of genes. Indeed, the H3K23 acetylation level of the *Ccp110* gene, as a representative example, which is involved in the regulation of cilium assembly, was decreased at the TSS by *Acly* knockdown (Fig. 6d). We verified the ACLY-dependent H3K23 acylation occupancy at the *Ccp110* promoter by ChIP-qPCR. As expected, a significant reduction in H3K23Ac, H3K23Bu, H3K23Cr, and H3K23Pr was induced by *Acly* knockdown at the “B” region compared to the negative control “A” region (Fig. 6e). Altogether, our data indicate that a subset of genes that is particularly sensitive to the integrity of the Ac-CoA pools may underlie epigenome remodeling for metabolic adaptation.

## Discussion

Mammalian cells have a remarkable ability to respond to diverse nutrient environments by rapidly changing gene expression. Furthermore, many cellular metabolites and metabolic enzymes are directly involved in the regulation of nuclear gene expression^5–7^. However, how cells manage these complex metabolic adjustments through the regulation of specific genes remains unknown. In this study, we revealed that acyl modification of H3K23 intimately mirrored the intracellular metabolic states. Fluctuating levels of Ac-CoA pools were reflected in the global loss or gain of H3K23 acylation marks and led to adaptive reprogramming of gene expression by simultaneously turning off a subset of genes involved in high-energy cellular functions while turning on other sets of genes involved in sustained survival. Our study shows that histone acylation acts as an immediate and reversible metabolic sensor enabling cellular adaptation to metabolic stress by reprogramming gene expression.

Acyl modification of histones neutralizes the positive charge of lysine residues and can affect chromatin structure and genomic function. However, the function of acyl modifications, including butyrylation, crotonylation, and propionylation, remains largely unknown. Crotonylation has been reported to increase during spermatogenesis and has been associated with active transcription^15,17,41^. Butyrylation and propionylation were identified as active chromatin marks that can stimulate transcription. Several ChIP-seq studies have revealed that H3K18Cr, H3K14Pr, and H3K14Bu are enriched in active chromatin, overlapping with their acetylated counterparts^13–15^. Here, we analyzed the genome-wide distribution profiles of H3K23 acylation marks, showing that H3K23Ac, H3K23Bu, H3K23Cr, and H3K23Pr have a very similar genomic distribution, with peaks at TSSs. In addition, acyl modification correlated with gene activity. These results suggest that H3K23Ac, H3K23Bu, H3K23Cr, and H3K23Pr participate in transcriptional regulation to facilitate gene expression. Interestingly, these modifications were reversibly responsive to nutrient availability and perturbation of Ac-CoA pools, implying that histone butyrylation, crotonylation, and propionylation are also metabolically regulated and involved in gene regulation upon metabolic challenges.

The molecular function of each H3K23 acylation mark is not clear. However, p300 and SIRT1 have been proposed to be writers and erasers of H3K23Pr^8^, whereas BRD4 has been proposed to be a reader because its bromodomains (BD1 and BD2) can bind to H3K23Bu and H3K23Pr^26^. Recently, Yan et al. showed that impairment of H3K23 acylation might be linked to neurodevelopmental disorders and cancers^42^. In light of the roles of H3K14 and H3K18 acyl marks, H3K23 acylation is likely to have a similar transcriptional regulatory function^13–15^, although further investigation is required to precisely address functions that are unique to these acyl modifications.

Histone acetylation is metabolically driven by HATs that depend on Ac-CoA^4,5,7,32,43–47^. Therefore, one can assume a simple positive correlation between Ac-CoA level and global histone acetylation. In this study, we showed that the direct knockdown of metabolic enzymes involved in Ac-CoA/SCA-CoA synthesis reduced Ac-CoA abundance and the Ac-CoA/CoA ratio. Given that the catalytic activity of HATs such as p300 and CBP is regulated by autoacetylation of their regulatory loop^48^, reduced Ac-CoA might affect p300/CBP autoacetylation and acetyl-/acyl-transferase activity. Based on these findings, a low Ac-CoA level and reduced Ac-CoA/CoA ratio must have a key role in governing the levels of various histone acylation marks, including acetylation. In addition, it is clear from our data that a relative increase in regional histone acylation was evident at positively regulated genes, even under Ac-CoA-limited conditions, which further suggests that HAT-dependent histone acylation likely occurs at the level of individual genes and is not determined by the average cellular concentration of Ac-CoA.

Interestingly, our GO enrichment analysis of commonly affected genes under three different knockdown conditions revealed a unique subset of genes involved in cilium assembly and morphogenesis and DNA transcription as genomic regions sensitive to Ac-CoA pools. Primary cilia are nonmotile organelles protruding from the cell surface^49^. They function as signaling centers, similar to antennas, for embryonic development and organogenesis in diverse cell types and tissues. In particular, cilia assembly and disassembly have been reported to play critical roles in cell proliferation, Hedgehog signaling, and the differentiation of skeletal muscle cells^50^. As DNA transcription and cilium pathways are highly modulated by metabolic enzymes, these biological functions might be tightly regulated by metabolic support. Whereas, diverse genes were additionally changed under each knockdown condition, suggesting that cells might regulate the expression of different gene sets upon metabolic stress according to the metabolic role of each enzyme. The next challenge is to identify metabolic circumstances that might reveal the distinctive roles of different acyl histone modifications. Furthermore, it would be beneficial to understand the mechanism how histone acylation induces specific gene expression changes.

## Supplementary information

Supplemental information
