# Supplementary material for: Histone acylation marks respond to metabolic perturbations and enable cellular adaptation
Source: Exp Mol Med. 2020 Dec 11;52(12):2005–19. doi: 10.1038/s12276-020-00539-x (PMC8080766; doi:10.1038/s12276-020-00539-x)
Supplement: Supplementary file 1 — Supplemental information [file 12276_2020_539_MOESM1_ESM.pdf]

Histone acylation marks respond to the metabolic perturbations for cellular adaptation

Chanhee Jo[1], Seokjae Park[2,3], Sungjoon Oh[2,3], Jinmi Choi[1,4], Eun-Kyoung Kim[2,3], Hong-Duk Youn[4], Eun-Jung Cho[1*]

[1]School of Pharmacy, Sungkyunkwan University, Suwon, Gyeonggi-do 440-746, Republic of Korea [2]Department of Brain and Cognitive Sciences, [3]Neurometabolomics Research Center, Daegu Gyeongbuk Institute of Science and Technology, Daegu 42988, Republic of Korea [4]National Creative Research Center for Epigenome Reprogramming Network, Seoul National University College of Medicine, Seoul 03080, Republic of Korea

[*]Correspondence should be addressed to E.-J. Cho; email: echo@skku.edu Tel: +82-31-290-7781, FAX +82-31-292-8800

Supplemental Figure S1. Jo et al.

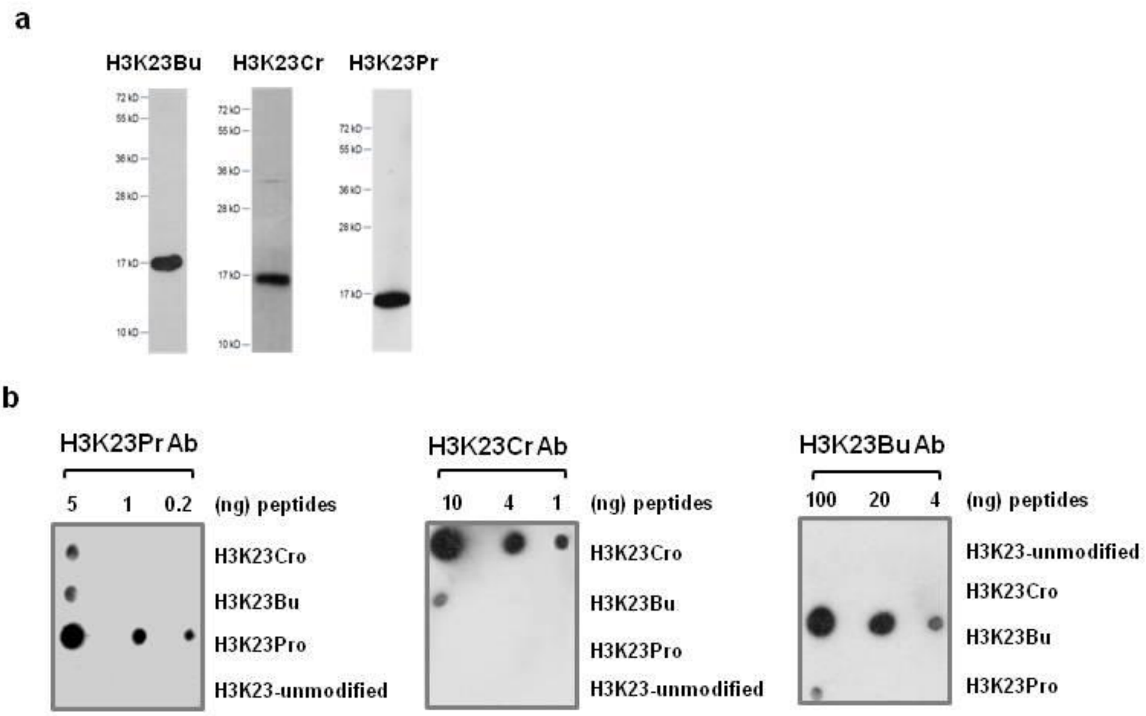

Supplemental Figure S1. Generation of H3K23 acylation-specific antibodies. (a) WB of 30 μg of HeLa whole cell lysates using H3K23Pr, H3K23Cr, and H3K23Bu antibodies. (b) Dot blot analysis of the indicated amount of peptides using indicated antibodies; final concentration, 0.5 μg/mL.

Supplemental Figure S2. Jo et al.

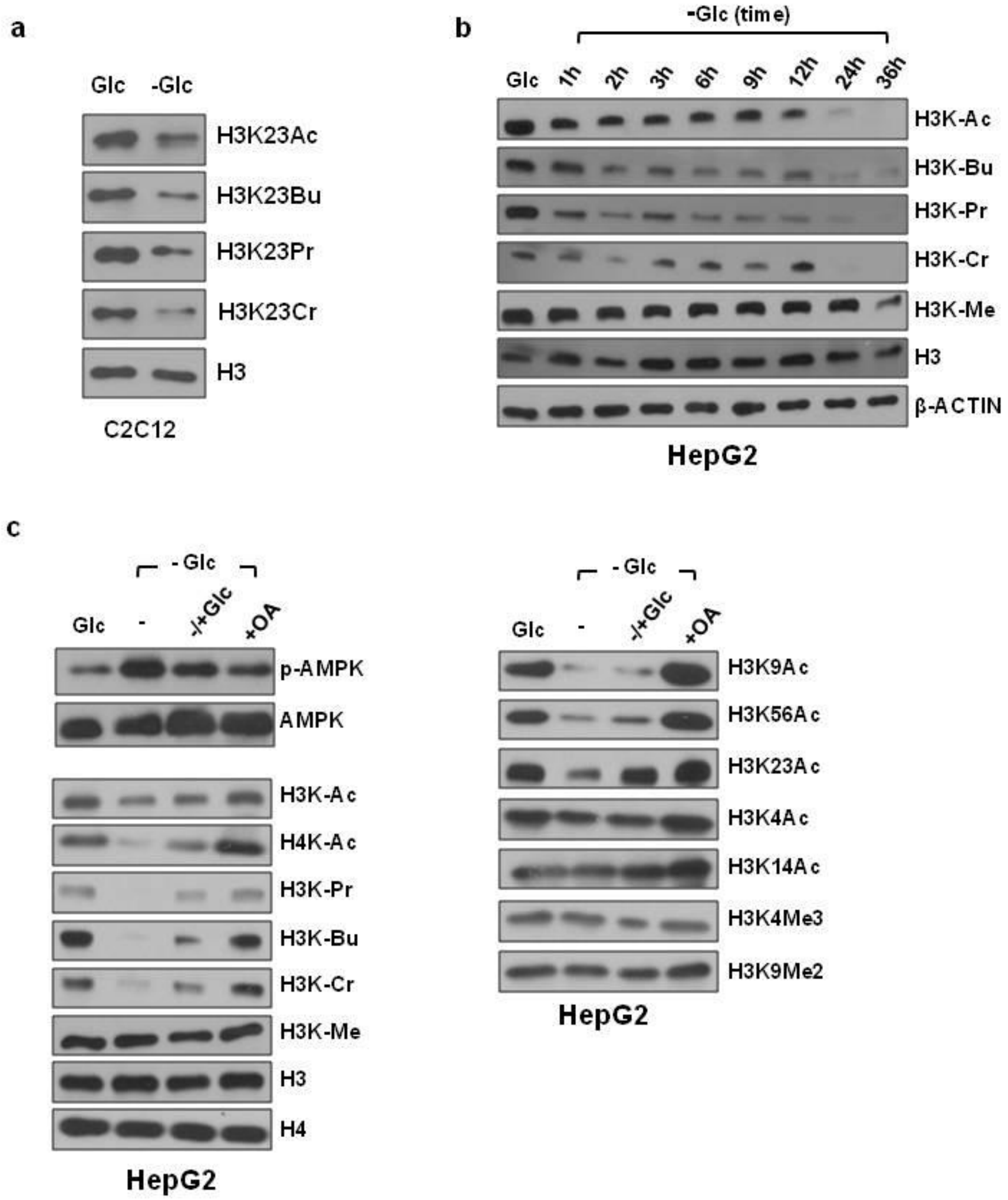

Supplemental Figure S2. Global histone acylation is affected by glucose and fatty acid. (a) WB of H3K23 acylations in C2C12 myotubes grown in "Glc", high glucose-DMEM; "-Glc", no glucose-DMEM for 24 h. (b) WB of cellular proteins of HepG2 cells grown in glucose-deficient culture medium for the indicated durations. (c) WB of cellular proteins of HepG2 cells grown in indicated culture conditions. Cells were cultivated in "Glc", high glucose DMEM for 24 h; "-Glc", no glucose DMEM for 24 h with fatty acid-free BSA; "-/+Glc", no glucose DMEM for 24 h followed by high glucose DMEM for 12 h; "+OA", no glucose DMEM for 24 h with oleic acid (OA)-conjugated BSA (200 μM of oleic acid).

Supplemental Figure S3. Jo et al.

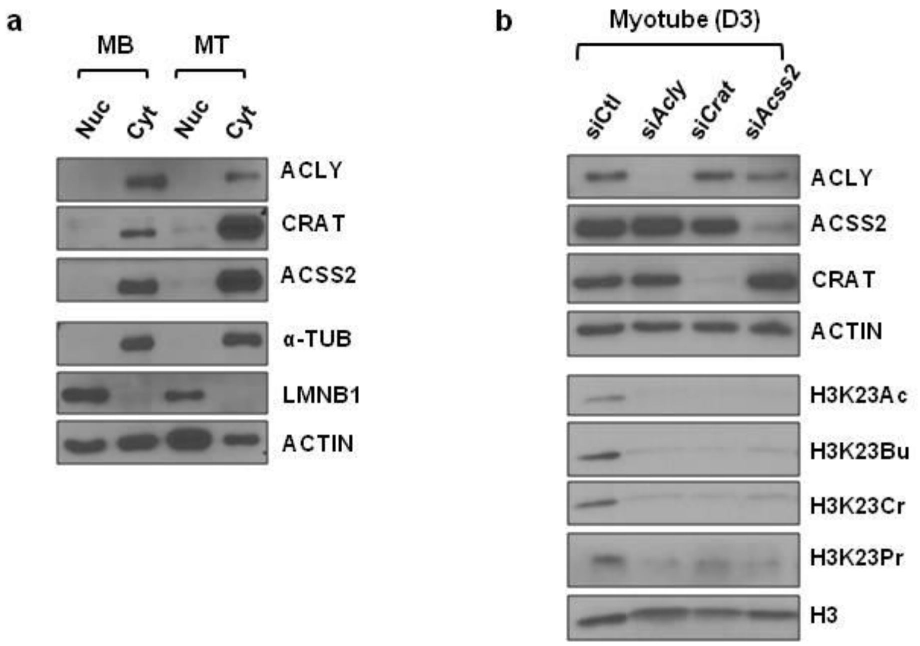

Supplemental Figure S3. ACLY, CRAT and ACSS2 are localized in the cytosol, and are required for H3K23 acylation. (a) WB of nuclear and cytosolic fractions of C2C12 myoblasts (MB) and myotubes (MT). Cellular localization of ACLY, CRAT, and ACSS2 protein was analyzed, showing that these enzymes were predominantly localized in the cytoplasm of both myoblast and myotube C2C12 cells. (b) WB of H3K23 acyl modifications in C2C12 myotubes. Myoblast cells were transfected with indicated siRNAs and differentiated for 3 days.

Supplemental Figure S4. Jo et al.

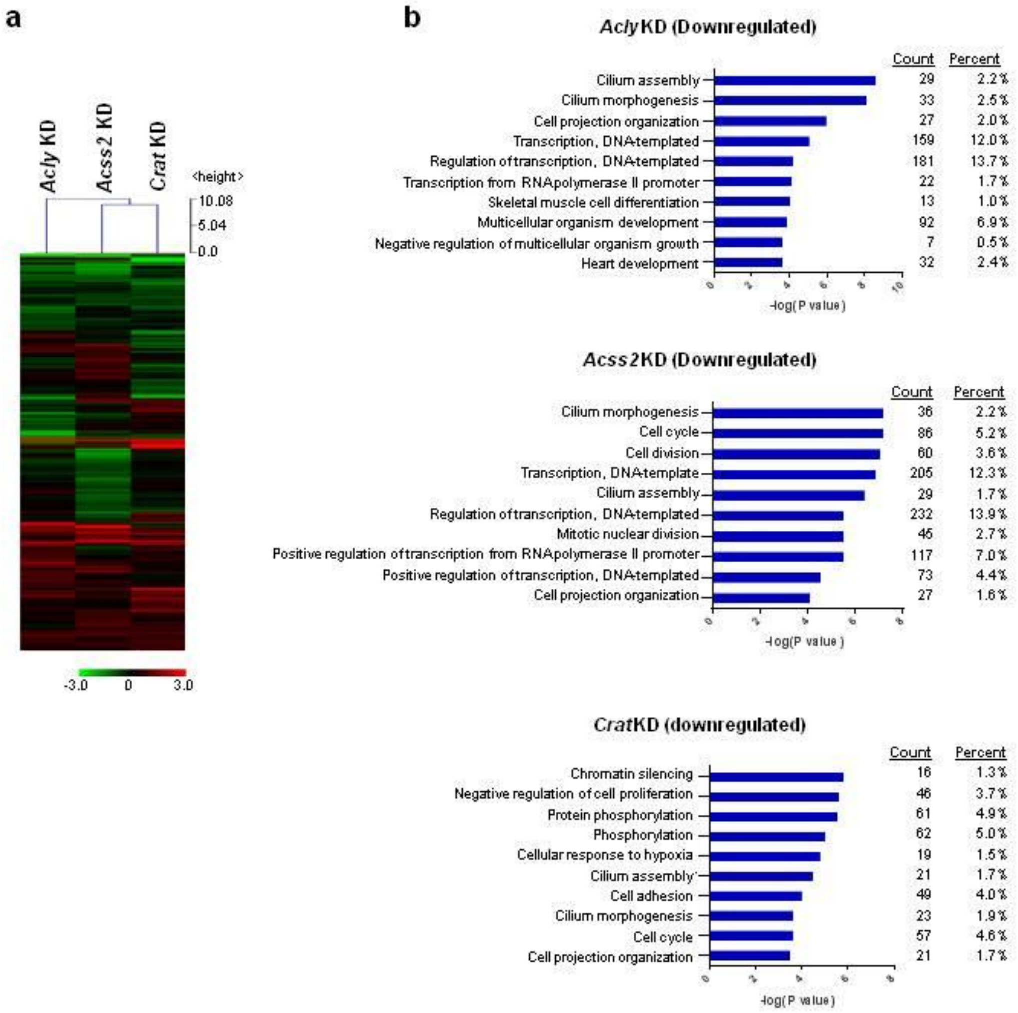

Supplemental Figure S4. Transcriptome analysis of *Acly*, *Crat* and *Acss2* KD. (a) Heatmap of all expressed genes in *Acly*, *Acss2,* and *Crat* KD C2C12 myoblasts using RNA-seq data (relative to siCtl, |FC| ≥ 1.5). (b) DAVID GO analysis using significantly upregulated gene sets (|FC| ≥1.5). The top 10 ranked biological functions are shown in order of ascending p-value. Each siRNA KD sample was compared with siCtl.

Supplemental Figure S5. Jo et al.

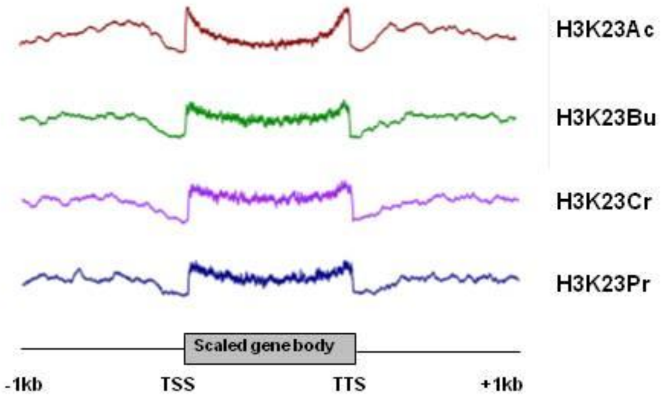

Supplemental Figure S5. Total metagene profile of normalized coverage of the indicated histone acylations. Gene bodies were scaled to have an equal length. C2C12 myoblast cells were analyzed by ChIP-seq with indicated H3K23 acyl modification-specific antibodies.

Table 1. Antibodies used in this study:

ANTIBODIES	SOURCE	IDENTIFIER
Rabbit polyclonal anti-H3Ac	Millipore	Cat#06-599
Rabbit polyclonal anti-Pan butyryl-lysine	PTM bio	Cat#PTM-301
Rabbit polyclonal anti-Pan propiopnyl-lysine	PTM bio	Cat#PTM-201
Rabbit polyclonal anti-Pan crotonyl-lysine	PTM bio	Cat#PTM-501
Rabbit polyclonal anti-H3	Abcam	Cat#ab1791
Rabbit polyclonal anti-H3K9Ac	Cell signaling	Cat#9671
Rabbit polyclonal anti-H3K56Ac	Active motif	Cat#39281
Rabbit monoclonal anti-H4Ac (clone 62-141-13)	Millipore	Cat#05-858
Rabbit polyclonal anti-H3K23Ac	Active motif	Cat#39131
Rabbit polyclonal anti-H3K23Bu	PTM bio	Custom-made Lot# PZ016C0728P059
Mouse polyclonal anti-H3K23Cr	PTM bio	Custom-made Lot# P Z022C1210M365
Rabbit polyclonal anti-H3K23Pr	PTM bio	Custom-made Lot# P Z017C0910P065
Rabbit polyclonal anti-H3K4Ac	Active motif	Cat#39381
Rabbit polyclonal anti-H3K4me	Upstate	Cat#07-436
Rabbit polyclonal anti-H3K9me2	Upstate	Cat#07-441
Rabbit polyclonal anti-Lamin B1	Abcam	Cat#ab16048
Rabbit polyclonal anti-ATP-Citrase Lyase (ACLY)	Cell signaling	Cat#4332
Rabbit polyclonal anti-Carnitine Acetyltransferase (CRAT)	Cloud-clone corp	Cat#PAC400Mu01
Rabbit monoclonal anti-AceCS1 (clone D19C6) (ACSS2)	Cell signaling	Cat#3658
Mouse monoclonal anti-actin (clone C4)	Millipore	Cat#MAB1501
Rabbit polyclonal anti-Pan methyl-lysine	Abcam	Cat#ab7315

Mouse monoclonal anti-flag (clone M2)	Sigma	Cat#F3165
Rabbit polyclonal anti-alpha tubulin	Abfrontier	Cat#LF-PA0146
Rabbit monoclonal anti-phospho-AMPKα (Thr172) (Clone 40H9)	Cell signaling	Cat#2535
Rabbit polyclonal anti-AMPKα	Cell signaling	Cat#2532

Table 2. siRNAs and primer sequences used in this study:

NAME	SOURCE	SEQUENCE or IDENTIFIER
control siRNA	Bioneer	SN-1003
si*Acly*	Bioneer	CAG CAA AGA UGU UCA GUA ATT
si*Crat*	Bioneer	CCA AGA AAC UGG UGG AUG ATT
si*Acss2*	Bioneer	ACA AAU ACA AGG UGA CCA ATT
Ccp110 ChIP primer region A	Bioneer	Fw: 5'-AATAAGCACCAGGAGGACGG-3'<br>Rv: 5'-CTTTGCAGATCAGTTGCGGG-3'
Ccp110 ChIP primer region B	Bioneer	Fw: 5'- GCGCTCTGTCTAAACCGACT-3'<br>Rv: 5'- GGGTCTCACCTCAGCCTAGA-3'
